# Advancing Profiling Sensors with a Wireless Approach

**DOI:** 10.3390/s121216144

**Published:** 2012-11-22

**Authors:** Alex Galvis, David J. Russomanno

**Affiliations:** Department of Electrical and Computer Engineering, Purdue School of Engineering and Technology, Indiana University-Purdue University Indianapolis, Indianapolis, IN 46202, USA; E-Mail: agalvis@iupui.edu

**Keywords:** wireless profiling sensor, neural network, object classification, sparse detector array

## Abstract

The notion of a profiling sensor was first realized by a Near-Infrared (N-IR) retro-reflective prototype consisting of a vertical column of wired sparse detectors. This paper extends that prior work and presents a wireless version of a profiling sensor as a collection of sensor nodes. The sensor incorporates wireless sensing elements, a distributed data collection and aggregation scheme, and an enhanced classification technique. In this novel approach, a base station pre-processes the data collected from the sensor nodes and performs data re-alignment. A back-propagation neural network was also developed for the wireless version of the N-IR profiling sensor that classifies objects into the broad categories of human, animal or vehicle with an accuracy of approximately 94%. These enhancements improve deployment options as compared with the first generation of wired profiling sensors, possibly increasing the application scenarios for such sensors, including intelligent fence applications.

## 1. Introduction

The concept of a sparse detector array profiling sensor, first proposed by R. Sartain from the U.S. Army Research Laboratory, utilizes a crude image (silhouette or profile) produced by a sparse detector array to classify passing objects as a means of surveillance in remote locations [1]. A vertical detector array, consisting of sixteen pairs of transmitters and reflectors, was first developed to produce sixteen parallel N-IR beams. A silhouette was then generated if the object broke the sensor’s beams [1–5].

Traditional cameras, including infrared imaging sensors, use a dense focal-plane array to capture the entirety of a given field of view (FOV), whereas a profiling sensor uses a sparse detector array to cover limited sampled sections of a FOV. The reduced data sensed from the FOV limits the types of conclusions that can be drawn about the sensed objects. Nevertheless, the types of conclusions that can be reliably deduced may satisfy the majority of the requirements of intelligent fence and analogous applications.

Moreover, profiling sensors are easily deployable on a large scale and are often regarded as disposable devices in applications that require surveillance of large, remote areas, which are often in hostile environments. Because of the relatively sparse number of sensing elements, these sensors are less expensive and produce a two-dimensional profile or silhouette of zero-dimensional samples. Profiling sensors are ideal for applications that require object classification using inexpensive, unmanned surveillance networks because of their low-cost components and efficient classification algorithms [6].

N-IR profiling sensors have been used in experimental data-to-decision ontology-based proof of concept frameworks, as well as in proposed sensor fusion schemes [7,8]. Also, profiling sensors have been used in a conceptual framework for detecting human intent from external stimuli [9].

One application of profiling sensor technology that has received increased attention is intelligent fence applications [10], including security and surveillance of the United States (U.S.) and other areas. The U.S. has approximately 12,000 kilometers of border, with numerous isolated and unpopulated sections. To improve the security of these borders by reducing illegal activities, improved surveillance is a top priority. One approach to these challenges has been the research and development of low-cost profiling sensors that can reliably discriminate among humans, animals, and vehicles. Profiling sensors can be used as surveillance devices to monitor trails and small unguarded roads, for example, border areas between the U.S. and Mexico that are used for illegal trafficking and smuggling of contraband. Reliably classifying objects that traverse these routes is of high interest in defense and homeland security applications [6,11–13].

Currently, it is not economically feasible to monitor an area as extensive as the U.S. and Mexico border due to the cost and maintenance associated with traditional, high-cost imagers and other sensing devices. The Department of Homeland Security (DHS), through the SBI.net project, is the pioneer in the development of major border security devices for highly trafficked areas [14]. Infrared cameras and moving target indication (MTI.) are examples of advanced sensing equipment that can operate in a network-centric environment to track human activity across a boarder. However, when completed, the deployment of such equipment along the U.S. boarder is estimated to be $620K per km (approximately $1 million dollars per mile of border) [15]. With the excessive expense for full surveillance coverage using the SBI.net project system, the low-cost alternative of a profiling sensor network is of interest, either as a complementary or competing technology.

In the previous work of Galvis *et al*. [16], a wireless N-IR profiling sensor was first introduced as an advancement to profiling sensors. This paper extends that prior work and presents the wireless profiling sensor in a broader context. The sensor presented in this paper incorporates a wireless sensing element, a distributed data collection and aggregation scheme, an enhanced classification technique, as well as other hardware improvements to advance profiling sensors. These enhancements improve deployment options as compared with the first generation of wired profiling sensors [17], possibly increasing the application scenarios for such sensors.

This article also includes a review of representative work in profiling sensors, specifically the foundational research and development that led towards designing and building the wireless prototype. Section 2 presents the assembly, data acquisition, and associated software of the wired and wireless profiling sensors. Section 3 highlights some alternative approaches to realizing profiling sensors. Lastly, Section 4 provides conclusions and recommendations for future work.

## 2. Profiling Sensors that Use a Sparse Detector Array

A sparse detector array has been used in several previous profiling sensor prototypes to create a crude image, via a sparse binary matrix, which is converted into a silhouette. Using classification algorithms, these silhouettes can be identified as belonging to one of several predefined object classifications. The following sections will highlight the major advances of wired and wireless profiling sensors that use a sparse detector array. Section 2.1 will briefly discuss the advances in the family of wired profiling sensors. Furthermore, to highlight the latest work completed with N-IR profiling sensors, three major contributions of the wireless profiling sensor are discussed in detail in Section 2.2 along with the challenges this new prototype presents.

### 2.1. Wired Profiling Sensor

**Figure 1.**
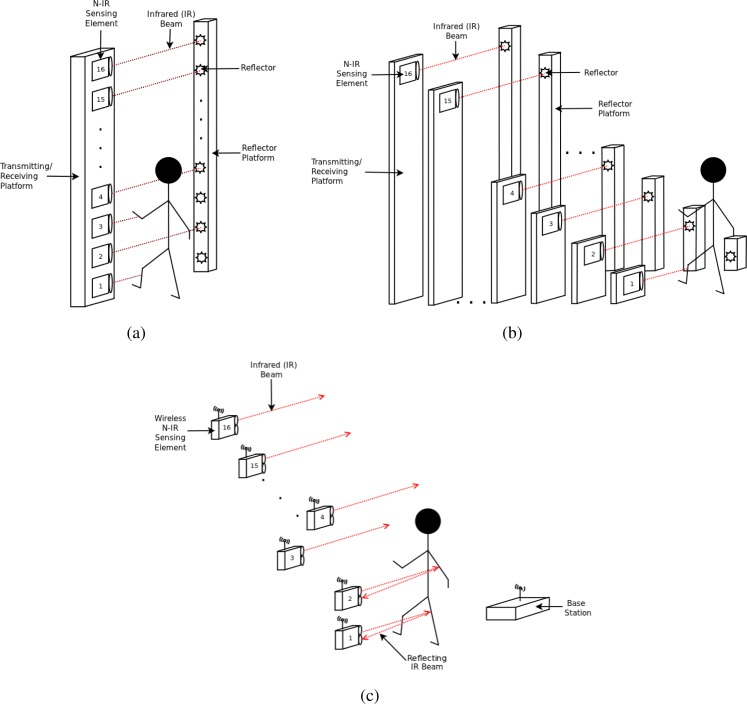
N-IR profiling sensor. (**a**) Sparse vertical array of wired detectors; (**b**) Sparse vertical array of wired detectors with custom horizontal offset; (**c**) New wireless profiling sensor comprised of sixteen N-IR sensing elements. The aggregation of these beams comprises the wireless sensors FOV. The sensing elements or sensor nodes can be arranged in a myriad of configurations, enhancing deployment options.

The original sparse detector N-IR profiling sensor prototypes were built using a collection of sixteen pairs of transmitters and reflectors to produce sixteen parallel N-IR beams. The first prototype of the profiling sensor consisted of a vertical arrangement of these transmitters and receivers mounted on a platform consisting of two separate poles. The transmitters were mounted on the first pole and create a N-IR beam that is perpendicular to the paired reflecting surface mounted on the opposite pole (Figure 1(a)). In this configuration, the vertical profiling sensor has been shown to successfully generate and accurately classify silhouettes among predefined classification groups [1–5]. For this initial prototype, there was a zero horizontal offset between the transmitters and receivers. In laboratory practice, this arrangement of transmitters and receivers reduces the amount of processing necessary to generate a crude silhouette since no further realignment algorithms are needed before classification algorithms are executed. However, a vertical array of detectors with no horizontal offset induces deployment constraints that limit applications in several instances. A second iteration of profiling sensor was developed with the capability to horizontally offset the detectors, providing additional deployment flexibility for such sensors.

The modifications in the second prototype not only supported horizontal offsets of the detectors, but also improved off-line data collection of the device. The second prototype used the same sensing elements, transmitters, and reflecting surfaces as the original vertical array prototype but supported a modified detector configuration with horizontal offsets between sensing elements (Figure 1(b)) [18,19]. Additional hardware improvements were made to eliminate the need of a computer in the field for data collection. An embedded microcontroller, Digi International BL4S200 single-board-computer, was used to illustrate that collecting, storing, realigning and classifying data could be performed by a low-resource device. In addition, a hand-held interface I/O box, developed by Reynolds *et al*. [19], facilitated data access through a microcontroller by allowing view access of classification results in the field.

#### 2.1.1. Data Acquisition

**Figure 2.**
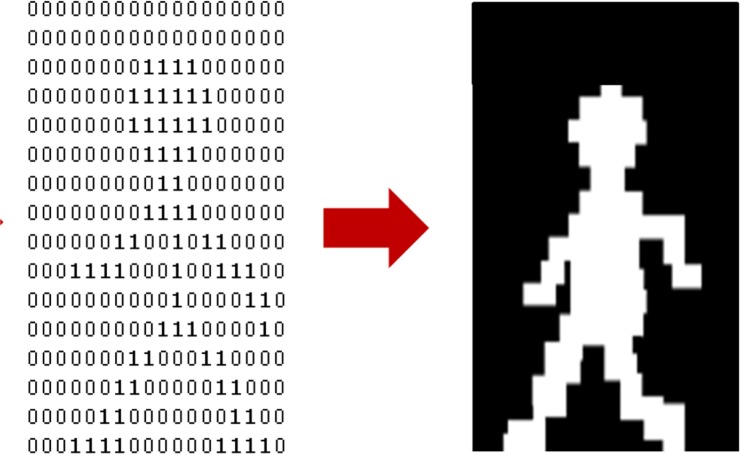
Resulting binary matrix data and silhouette from an object passing through the N-IR beams of a vertical array profiling sensor.

The sixteen sensing elements in the profiling sensors in Figure 1(a) and 1(b) are a set of parallel infrared beams that produce a series of “on” and “off” events that are respectively recorded as binary 1’s and 0’s into a matrix, which is then interpreted as a silhouette (Figure 2). Since each sensing element records these events using a fixed sampling rate, the data acquired for a slow passing object produces longer binary strings than for a faster moving object [19–21]. For instance, a human would generally produce longer strings than a vehicle, since the vehicle is more likely to move at a higher velocity. Therefore, the resulting raw data can be represented as a *M* × *n* binary matrix, where *M* (*M* = 16 in most prototypes) is the number of sensing elements in the vertical sparse array, each row representing the binary data of one sensing element over the acquisition time. The number of columns *n* in the binary matrix is variable because the length of the string depends upon the speed of the object traversing the sensor’s FOV. The binary matrix used to generate a raw silhouette image of the object is also used as input for subsequent classification processing.

**Figure 3.**
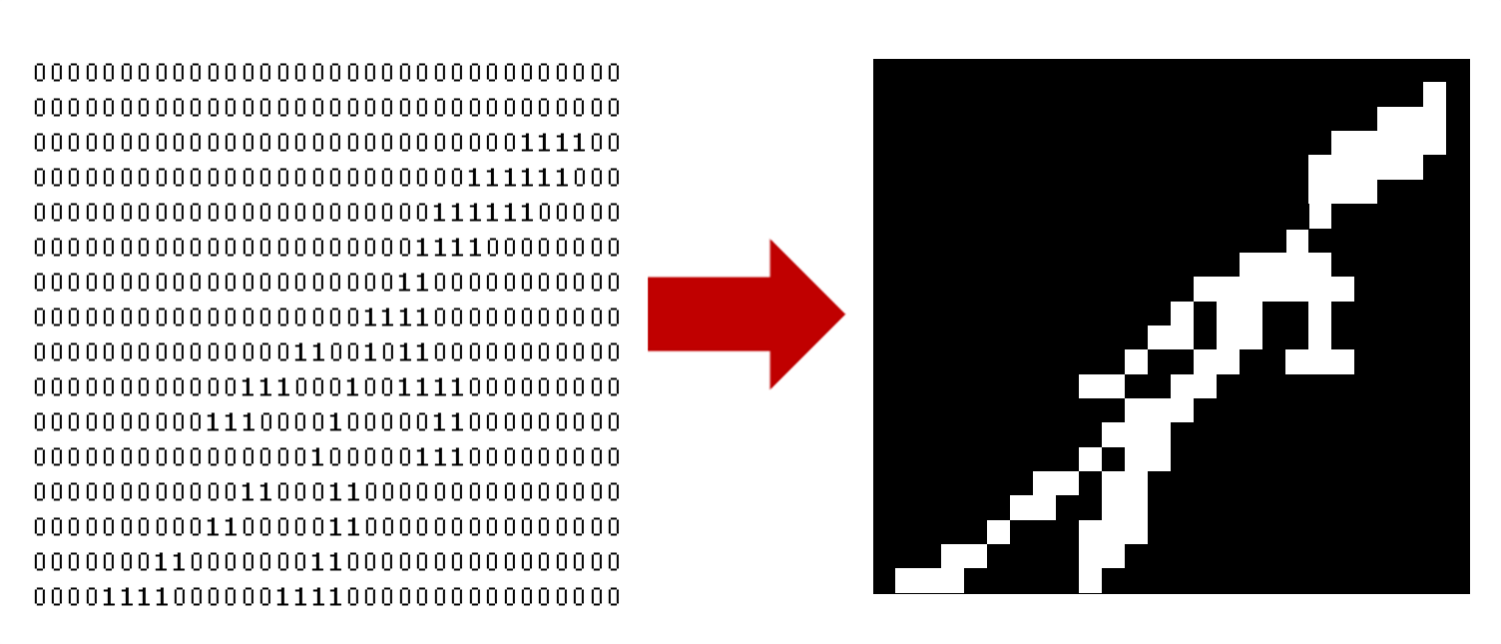
Resulting binary matrix data and silhouette from an object passing through the N-IR beams of a custom array profiling sensor.

**Figure 4.**
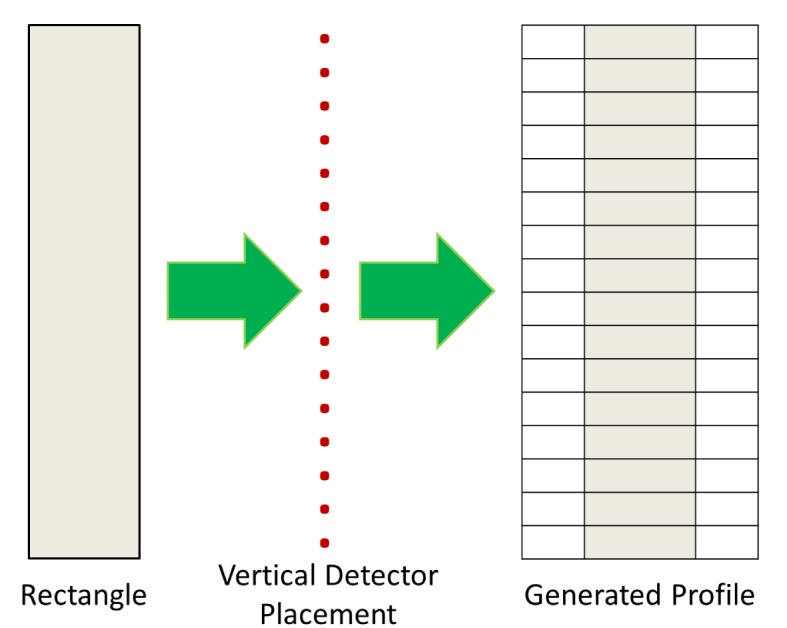
Vertically straight rectangle passing through vertical array N-IR profiling sensor.

The profiling sensor prototype with horizontal offsets generates an analogous matrix. However, the offset detectors or sensing elements introduce a distortion if the silhouette is processed as in the vertical detector case (Figure 3). To explain this distortion further, consider a vertical rectangle moving at constant velocity across the FOV of the vertical profiling sensor. Intuitively, the binary silhouette generated by the vertical system should look as shown in Figure 4, given that there is no horizontal offset of detectors. However, applying the same algorithm to data acquired from a profiling sensor with horizontally offset detectors causes the system to record blank data (0’s) from sensing elements where the infrared beam is unbroken while the object reaches the next detector. The raw image is expressed as shifted pixels with the gap size proportional to the horizontal distance between the detectors.

**Figure 5.**
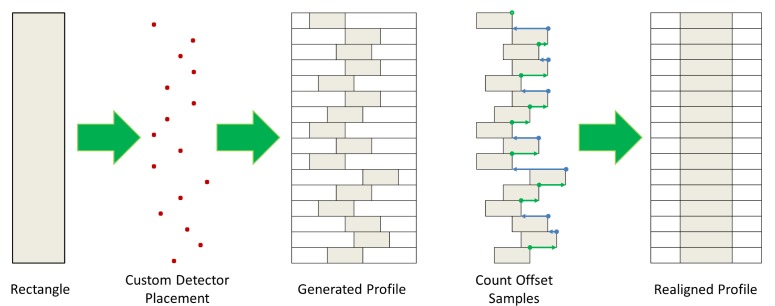
Vertically straight rectangle passing through custom array N-IR profiling sensor before and after realignment algorithm.

With further processing necessary for the desired image production and classification, an algorithm was developed to correct for the horizontal offset caused in the data by the new configuration. The first step was a required calibration. For this process, a vertical rectangle moving at approximately constant speed is passed across the detectors. By counting the samples between the initial trigger, when the leading edge of the rectangle breaks the beam of the first detector in the array, and the next trigger, the offset can be calculated and used to realign the data collected from the next detector in the array relative to the first [18,19]. Likewise, the same technique can be used once the leading straight edge reaches subsequent detectors to realign their corresponding data relative to the first detector. Figure 5 shows how the silhouette corresponding to a traversing object can be realigned using this method.

Using the same technique to count the samples between each detector for data realignment, the velocity of a passing object can be approximated in cases where there is a predetermined distance between the detectors [18]. To attain the average velocity of the object over the entire range of the sensor array, every inter-sensor velocity of the array must be calculated, not merely the velocity between adjacent sensing elements. The formula stated in Equation (1) expresses a two dimensional weighted average of the velocities across the entire array, where *i* and *j* are the initial and final sensing elements of interest, respectively, *v_ij_* is the velocity among elements calculated by Equation (2), and *M* is the number of sensing elements in the array [19,21]. Notice that the velocity indicated by Equation (2) is calculated by dividing the distance over the time it took the passing object to move from sensing element *i* to sensing element *j*. This weighted velocity approach can also be used in silhouette realignment, but notice that in this case it is necessary to have at least an approximate measured distance.

(1) $$\bar{v}=\frac{2}{M\left(M-1\right)}\sum_{i=1}^{M-1}\sum_{j=i+1}^{M}v_{ij}$$

(2) $$v_{ij}=\frac{d_{ij}}{t_{ij}}$$

Enabling the profiling sensor to run autonomously is one of the goals towards a ready-to-deploy system that can collect data without the need of a graphic user interface or other cumbersome hardware like a monitor, mouse, or keyboard [18]. A Rabbit® 4000 Low-EMI, High-Performance Microprocessor [22] has been introduced to make the profiling sensor operate offline without the need of personal computers. Even though a shortcoming of the Rabbit® microcontroller is its limited 60 Hz sampling rate, experimentation has shown that the lower sampling rate of the microcontroller is still sufficient to provide enough detail for accurate object classification or other processing [23]. Equation (3) is a one-dimensional sum that creates an 8-bit string by reading the on-off value of the sensing elements ported to the pins in the microcontroller. The variable *s_i_* is therefore the on-off value that is shifted to the appropriate position in the string relative to the sensing element’s vertical position *i* from the ground.

(3) $$P_{x}=\sum_{i=1}^{8}\left(s_{i}\times 2^{i-1}\right)$$

The sparse vertical detector array and the subsequent design with horizontally offset detectors provided a proof of concept that objects could be reliably classified by the silhouettes produced by these low-resource profiling sensors. A wireless version of such a sensor would improve deployment options and also facilitate the design of a network-centric profiling sensor environment. The third profiling sensor prototype focused on enhancing the horizontally offset version of the profiling sensor to a sustainable wireless model thereby increasing options for custom detector placement, which will increase deployment options in many applications.

### 2.2. Wireless Profiling Sensor

The wireless profiling sensor is the latest advancement in the category of N-IR profiling sensors. The wireless profiling sensor is comprised of sixteen wireless N-IR sensing elements, each emitting an IR beam across a designated trail (Figure 1(c)). Compared with the prior work in N-IR profiling sensors, there are three major advances with this new sensor. First, the sensor is wireless and it sends data to a remote base station. Second, it uses an alternative technology for its sensing elements, that is, its detectors. With previous N-IR profiling sensors, a collection of transmitter and receiver pairs were required to detect an obstructing object along the path of an emitting IR beam. In the enhanced wireless prototype, a sensing node was configured to include a sensing element that does not require a mounted reflecting surface to detect a broken or unbroken beam event. The third significant improvement is in the data transmission method. Unlike the previous models where continuous streams of data were collected, the sensing nodes of the wireless prototype produce a bit of data corresponding to a broken or unbroken beam that is transmitted to the base station only when a state transition occurs. One motivation of the wireless design was to produce a readily concealable device, while also making a first attempt toward more efficient power usage by improving the data collection method since minimizing battery consumption is a significant design constraint.

The following sections provide details about the hardware, assembly, and software developed for the wireless profiling sensor. Section 2.2.1 discusses the new sensing device used to realize the detectors. The assembly Section 2.2.2, provides an overview of how the wireless N-IR profiling sensor was assembled. Next, Section 2.2.3 explains the data acquisition by the time segmentation method to generate the binary matrices used to generate silhouettes. Finally, Section 2.2.4 summarizes the software developed to process the incoming data into binary matrices and their corresponding silhouettes.

#### 2.2.1. Sensing Element

**Figure 6.**
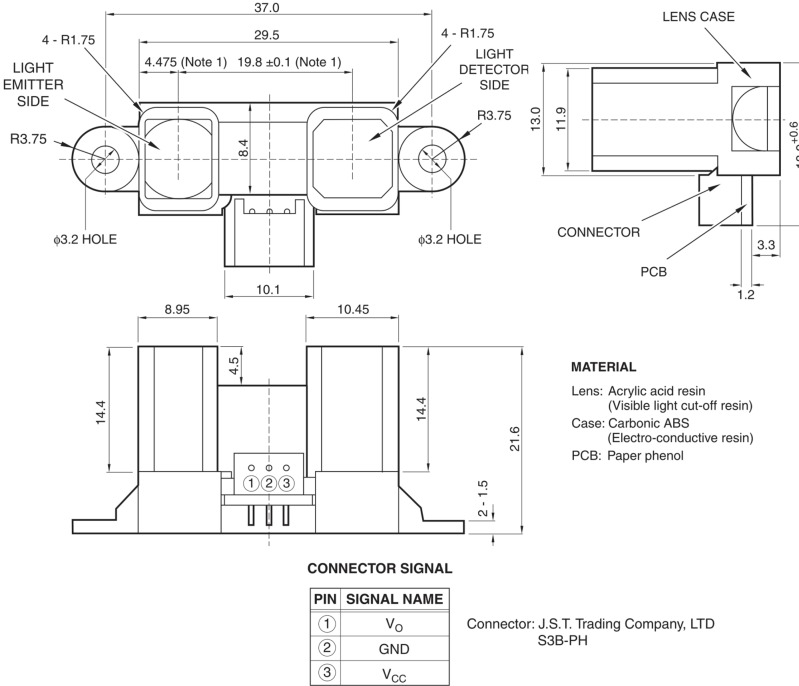
Sharp GP2Y0D02YK0F distance measuring sensor with integrated signal processing and digital output.

To make the wireless profiling sensor independent from a pairing reflective surface, the Sharp GP2Y0D02YK0F [24] (Figure 6), an N-IR distance sensing device, was used to detect objects along the path of each sensing element. The Sharp sensing element is a single point distance module, which measures the distance from the emitting infrared diode to the reflecting surface of the object.

**Figure 7.**
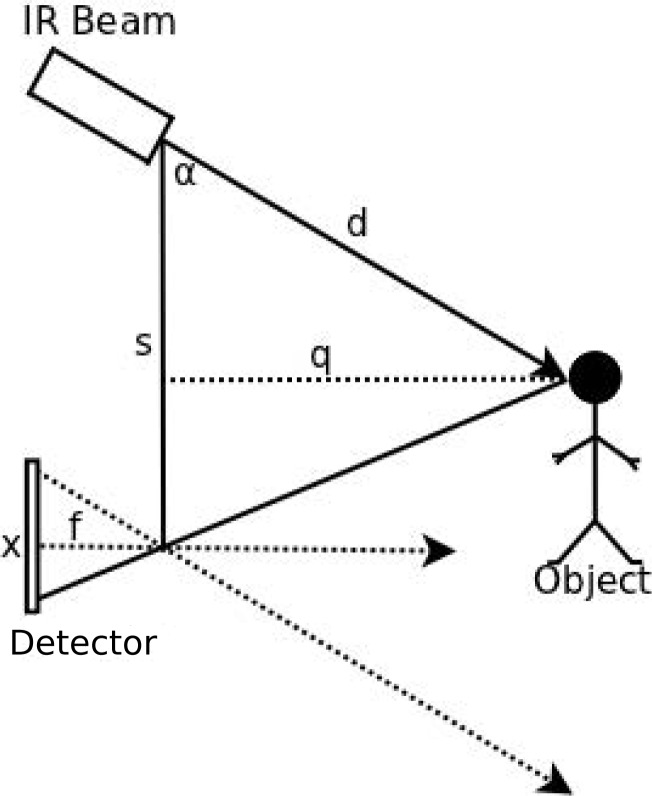
Relative distances and angle between object and individual elements in the distance sensor.

The effect of environmental light or the reflective properties of the sensed object on the GP2Y0D02YK0F is not significant up to a distance of approximately 80 cm. The N-IR distance sensor module utilizes the triangulation principle: the laser path is initiated at the N-IR beam emitter, reflects from the object, and is captured by the detector. When this event occurs, the output line of the sensing element is set to a logical high; otherwise, it is set to low. The relationship between the emitter, object, and detector is shown in Figure 7. The horizontal distance from the emitter to the object relies on the following: 1) the angle created between the emitted laser path to the object and the reflected laser path to the detector; and 2) the distance between the IR beam and the detector. The horizontal distance can be easily calculated by Equation (4), while Equation (5) utilizes the sine of the angle alpha to determine the horizontal distance [25].

(4) $$q=\frac{\left(f\right)\;\left(s\right)}{x}$$

(5) $$d=\frac{q}{\text{sin}\left(\alpha \right)}$$

#### 2.2.2. Assembly

**Figure 8.**
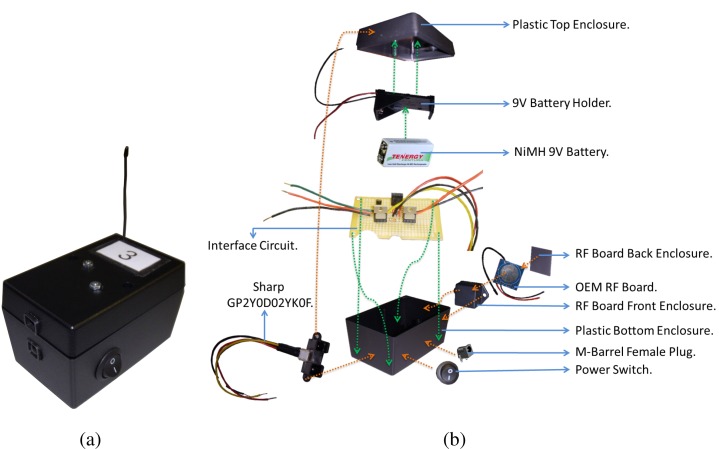
Wireless sensing node (**a**) Assembled view of one node; (**b**) Exploded view of one sensing node showing its internal components.

The wireless profiling sensor prototype is made up of a collection of sixteen wireless sensing nodes. An example of one of the sensing nodes is shown in Figure 8(a). Each sensing node is constructed from a combination of off-the-shelf components, including the Sharp GP2Y0D02YK0F sensing element (Figure 8(b)). Sixteen sensing nodes were used in the first wireless prototype to provide consistency with respect to prior N-IR profiling sensor designs and to facilitate comparisons with prior work. In practice, it is anticipated that the number of sensing nodes (*i.e.*, detectors) would be determined by the required probability of identification and tolerable false-alarm rate permitted for the sensed object by the application of interest. All sensing nodes in the wireless profiling sensor communicate with a remote computing device, such as a PC, via a USB gateway. The USB gateway and the components shown in Figure 8 are necessary for the basic function of the wireless N-IR profiling sensor. The node charging station and related cabling are non-essential accessory devices, but they improve the ease of use of the wireless profiling sensor in laboratory development scenarios, as well as in field tests.

**Figure 9.**
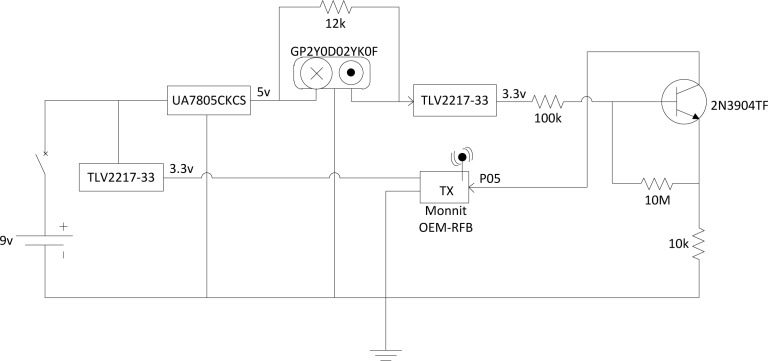
Circuit schematic of the internal component configuration of one wireless sensing node.

The signal regulator circuit divides the supply voltage to meet the specifications of the proximity sensor and wireless transmitter, using 5 V and 3.3 V of the original 9 V, respectively. To increase the power stability due to a discharging battery, a series of voltage regulators were incorporated into the design. A switching NPN transistor is used to close a circuit between the output of the proximity sensing element and the digital input of the wireless transmitter in order for the transmitter to detect when there is an infrared beam obstruction in the proximity sensor. In addition, a voltage regulator is placed in series with the base of the switching transistor to further regulate the digital output signal traveling from the proximity sensor to the transmitter. Figure 9 shows a schematic of this circuit design.

Since the profiling sensor is designed for deployment in a remote location, it was necessary to provide a power solution that would require little maintenance in a laboratory or field-test environment, albeit the sensor will be considered disposable for some anticipated applications. The power supply is a collection of off-the-shelf NiMH battery chargers, which resulted in a simplistic design, effectively eliminating the need for battery replacement. The focus during the design of this recharging station was on preservation of battery life. The charger includes a microprocessor that automatically controls the charging process. With this control feature, all batteries within the 10 channels are able to maintain a full charge without overcharging and causing battery damage. By choosing a charging station that only contained the needed features, the cost remained low for the power supply. The power supply contains two of these 10 channel recharging stations. For this design, only 16 of the channels are in use as the profiling sensor only requires the use of one 9 V battery per sensing node. The two recharging stations are housed in an ABS box for protection from the elements. Wires connect the output channels of the recharging station through an M-barrel connector for external access. The power supply draws power externally from one of two sources: the solar panel or a standard 60 Hz, 120VAC wall outlet. The power supply uses whichever connected source provides the highest potential once the latter is converted to 12VDC. This power supply box makes the recharging process for the wireless profiling sensor batteries convenient and efficient since it minimizes personnel time and optimizes battery life.

#### 2.2.3. Data Acquisition

To gain sufficient data to classify a passing object, the status of each node must only be known at two stages: when the object first obstructs the beam and when the beam is restored. Previous profiling sensor prototypes utilized a constant collection of data at a set frequency for its method of data acquisition [20,21]. As previously mentioned in Section 2.2.2, a similar data acquisition method would quickly drain the battery of the wireless profiling sensor since the transmitter needs to pull current for every packet transmission. Therefore, we have implemented a data acquisition method that not only uses the battery more efficiently, but only uses one data packet per change of state from each sensing element to generate the necessary raw data.

In the wireless profiling sensor, there is a designated trigger node that alerts the base station to the presence of an object. When this node detects an obstruction, the base station will begin recording data from all sensing nodes for a period of time specified in the sensor’s configuration setup. A packet contains the state of the node and the source node identification number. The state of the node is a binary code in which zero (0) indicates an unbroken beam and one (1) indicates an obstructed beam. As the base station collects data packets from the various sensing nodes that comprise one wireless profiling sensor, it will also record the time in milliseconds of when each packet is received. After the specified recording time expires, the base station ceases to record data. The recording of one object during this period of time is denoted as one recorded event. The base station sorts the collected transmission records of the event by source node identification numbers. Then an algorithm determines the time length of the recorded event by calculating the difference between the time stamp of the final packet received and the time stamp of the initial packet received. In previous prototypes, time stamps were unnecessary because of the continuous data recording, but since this new prototype collects data only when a change of state occurs, we must generate the frequency of data acquisition for our non-constant rate to develop a binary matrix representation of this data.

**Figure 10.**
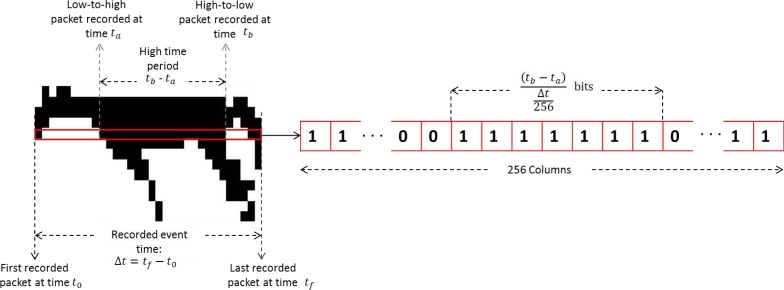
Populating a binary row by using a time segmentation method.

The resulting binary matrix is a 16 × *n* matrix in which every entry corresponds to a low or a high bit reading at a point in the sampling. To generate this matrix with the collection of time samples received, the total time length of the recording Δ*t* is calculated by subtracting the time of the first packet received *t*_0_ from the time the last packet is received, *t_f_*. Equation (6) is then used to find the length of a time segment that will eventually be replaced by a binary value corresponding to the node state in that time interval. Additionally, *t_b_* − *t_a_* is the time difference from a change of state within Δ*t*. As shown in Equation (7), *t_b_* − *t_a_* is divided by the result in Equation (6) so that this time interval can be interpreted as a sequence of bits describing the constant state of the (*t_a_*, *t_b_*) interval. Figure 10 is a visual representation of this procedure. Repeating this procedure for each sensing node generates the 16 necessary binary rows making up the entire binary matrix. Finally, this matrix is used to generate a silhouette of the sensed object, as well as input data to a back-propagation neural network for subsequent object classification.

(6) $$s=\frac{\Delta t}{256}$$

(7) $$N=\frac{t_{b}-t_{a}}{s}$$

#### 2.2.4. Software Interface

**Figure 11.**
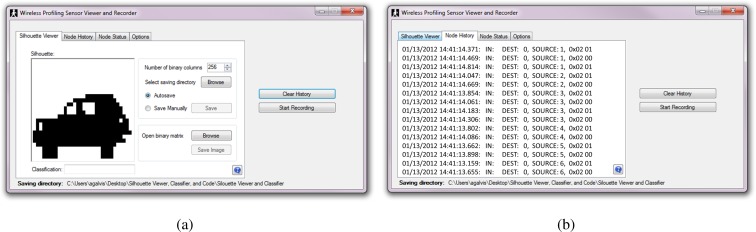
First two tabs of the silhouette viewer and classifier software (**a**) Silhouette viewer tab; (**b**) Node history tab.

**Figure 12.**
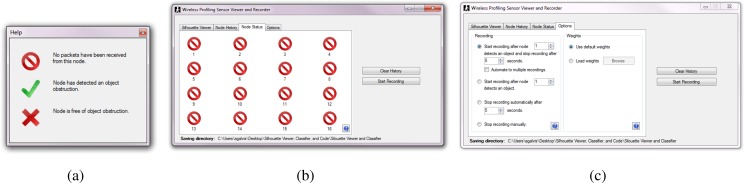
Second two tabs of the silhouette viewer and classifier software (**a**) Three possible node status display icons from the node status tab; (**b**) Node status tab; (**c**) Options tab.

Additional software was developed to support the development and test of the wireless version of the profiling sensor including the silhouette viewer and classifier. The software was divided into four different tabs, each providing a separate tool for the developer. The first tab, the silhouette viewer, displays silhouettes from previously recorded binary matrices, or from data being currently collected. Figure 11(a) provides a screenshot of the “Silhouette Viewer” tab. The second tab is the node history tab, which displays a list of node ID’s along with their current state (1-high, 0-low) and the time when each packet was received. Figure 11(b) shows a screenshot of the “Node History” tab. The third tab is the node status tab, which shows 16 icons corresponding to the current status of each sensing node. The status of each node can be one of three possible statuses: “No packet received”, “IR-beam obstructed”, or “IR-beam restored”. Figure 12(a) shows the list of icons that represent the different statuses listed in the node status tab shown in Figure 12(b). Finally, the options tab (Figure 12(c)) is divided into weights and recording options. The weights option allows the user to choose from a preloaded weights file or a different file from a newly trained neural network. The recording option lists the different ways available to record new data from the wireless profiling sensor. The user may choose to stop recording after a time limit, have a trigger node that activates the recording of data for a time frame, or manually start and stop each recording event. Once the user begins to record new data, the new recorded event is stored in the specified directory as three separate files:

- **Binary Matrix**: .txt containing the binary matrix of the recorded event.
- **Node Status**: .txt that stores a sorted version of what is displayed in the “Node History” tab. This node history is sorted by node ID.
- **Silhouette**: .bmp that displays the silhouette of the recorded event.

#### 2.2.5. Classification Algorithm

The purpose of the classification algorithm is to accurately classify the data acquired from the wireless profiling sensor into predefined classes, for example, classify the data as human, animal, or vehicle. Classification is achieved through the use of a three-layer back-propagation neural network. The output of the neural network classifies data into one of the three predefined classes of objects. For completeness, some neural network fundamentals are summarized in the following paragraphs before the network configuration and results are summarized in Section 2.2.6.

##### Neural network

A neural network can be thought as system capable of processing a series of inputs in vector form to produce one output vector. Additionally, a neural network is a collection of weighted connections and processing elements in which the weighted values pass values from the processing elements in the previous layer. A neural network can have multiple layers and different types of feeding patterns between processing elements. However, depending on the application, the more involved neural network topologies may be more difficult to train or may take more computational resources to process an input vector. Therefore, for simplicity, a three-layer back-propagation neural network (with feedforward pattern matching) has been used here as this produces an output corresponding to the closest estimation for the given input. Finally, a few definitions concerning neural network key elements and concepts are presented below as summarized from Eberhart and Shi [26]:

- **Network weights**: Regulates the amount of dataflow passing from one processing element to the next. Weight connections can be excitatory if its value is positive or inhibitory if it is negative. A weight with value of zero simulates an absence of connection between the processing elements.
- **Processing elements**: Neural network components where calculations are executed. A processing element can have single or multiple inputs that are passed down from other processing elements trough the network weights. Each processing element collects these local inputs to produce a single local output value. Additionally, these outputs can be the input to other processing elements or the output to the neural network.
- **Processing element activation function**: Maps the domain of a processing element to a specified range. There are infinitely many functions that can be used to perform this mapping; however, functions that bound the output to a fixed range are often desirable because these often reduce computation time during training. A common activation function of choice is the sigmoid function as it has a bounded range for any real argument.

Although the original raw data had varying column dimensions [19–21], when using a library of over 1000 silhouettes captured in the field [27], including humans, animals and vehicles, it has been shown that compressed data with a fixed number of columns produces virtually no alterations in the silhouette resolution [23]. Since a back-propagation neural network is used to classify the data, a fixed number of inputs were required to obtain a classification result. After normalizing the data to 256 columns, the back-propagation neural network was trained with half the data set within the compressed library. This neural network is comprised of 256 *×* 16 = 4,096 processing elements in the input layer, 20 processing elements in the hidden layer, and 3 processing elements in the output layer. This configuration was observed to provide the best classification results compared with other back-propagation neural networks with differing numbers of processing elements within the hidden layer. The method used to train the neural network was from Ebehart and Shi [26].

**Figure 13.**
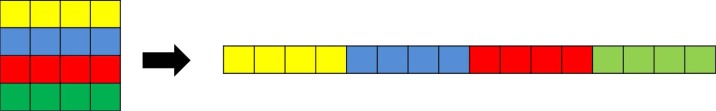
Matrix vectorization: All rows from a two-dimensional matrix are put sequentially into a linear array.

**Figure 14.**
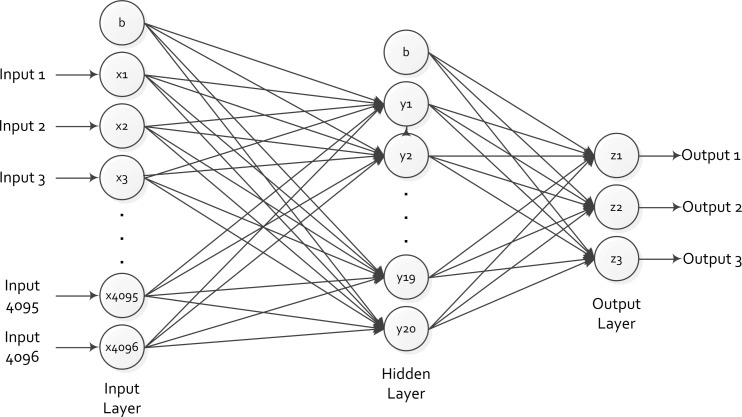
Back-propagation neural network with 4,096 input processing elements, 20 hidden processing elements, and 3 output processing elements. Bias processing elements are denoted by b and these take an input value of 1.

To make the matrix easier to process by the neural network the binary matrix has been vectorized by sequentially combining all rows (Figure 13). Each processing element in the input layer uses each bit entry in the vector matrix as the argument to a sigmoid function (Equation (8)), which will generate a real value between 0 and 1. The output values from each processing element in the previous layer become the input values for each of the processing elements in the subsequent layer. The edges connecting the processing elements from one layer to its subsequent layer have a weight value that is generated during the training process of the neural network. The weight of the edge is then multiplied by the input for each processing element in the hidden layer. The sum of these 4,096 products becomes the argument for the sigmoid function of the hidden layer. This process is repeated from the hidden layer to the output layer to generate three output values. A schematic of this neural network is shown in Figure 14. Equations (9) and (10) are used to calculate the input values to the processing elements in the hidden and output layer. The values *v_ih_* and *w_ji_* denote the weight values from the input to the hidden layer and from the hidden to the output layer, respectively, which are all generated during the training of the neural network. Furthermore, notice that the sum of products begin from *h* = 0 and *i* = 0, the bias processing elements, which take an input value of 1.

(8) $$f\left(x\right)=\frac{1}{1+e^{-x}}$$

(9) $$y_{ki}=f\left(\sum_{h=0}^{n}\left(\left(x_{kh}\right)\left(v_{ih}\right)\right)\right)$$

(10) $$z_{ki}=f\left(\sum_{i=0}^{h}\left(\left(y_{ki}\right)\left(w_{ji}\right)\right)\right)$$

#### 2.2.6. Results

Prior work with the first generation, sparse vertical detector array N-IR profiling sensors included a significant effort to build a comprehensive library of sensed objects, including numerous vehicles (e.g., motorcycles, cars, trucks, SUVs, ATVs, *etc*.), humans (e.g., men and women of various sizes and gaits, and carrying various loads, including backpacks and weapons), and animals (e.g., dogs, horses, llamas, *etc*., including carrying various loads) [27]. Data corresponding to approximately 580 sensed objects from this comprehensive pre-existing library was used to train a new back-propagation neural network to classify objects sensed by the new wireless version of the profiling sensor. An additional 577 sensed objects from the pre-existing library was used to test the accuracy of the newly trained neural network. The number of human, animal, and vehicle samples used in both training and testing of the back-propagation neural network are shown in Table 1. Having a pre-existing data library of animals, humans, and vehicles expedited the training and testing of the neural network to be used in the new wireless sensor. A very small library of sensed human silhouettes acquired directly from the new wireless N-IR profiling sensor was also used to test the performance of the neural network trained with the pre-existing library. These results from data acquired directly from the wireless sensor are briefly summarized after the results when using the pre-existing library.

**Figure 15.**
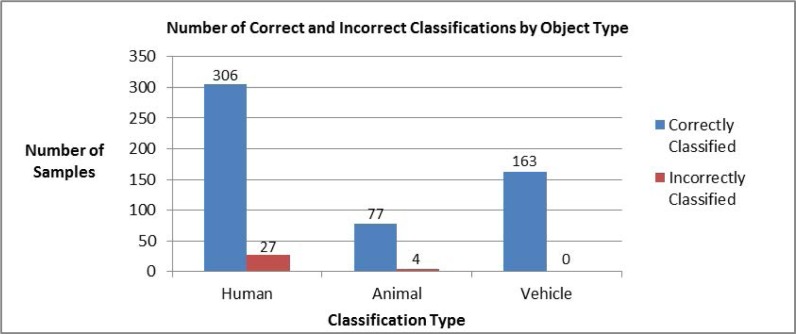
Distribution of number of samples per class within the testing data set.

**Figure 16.**
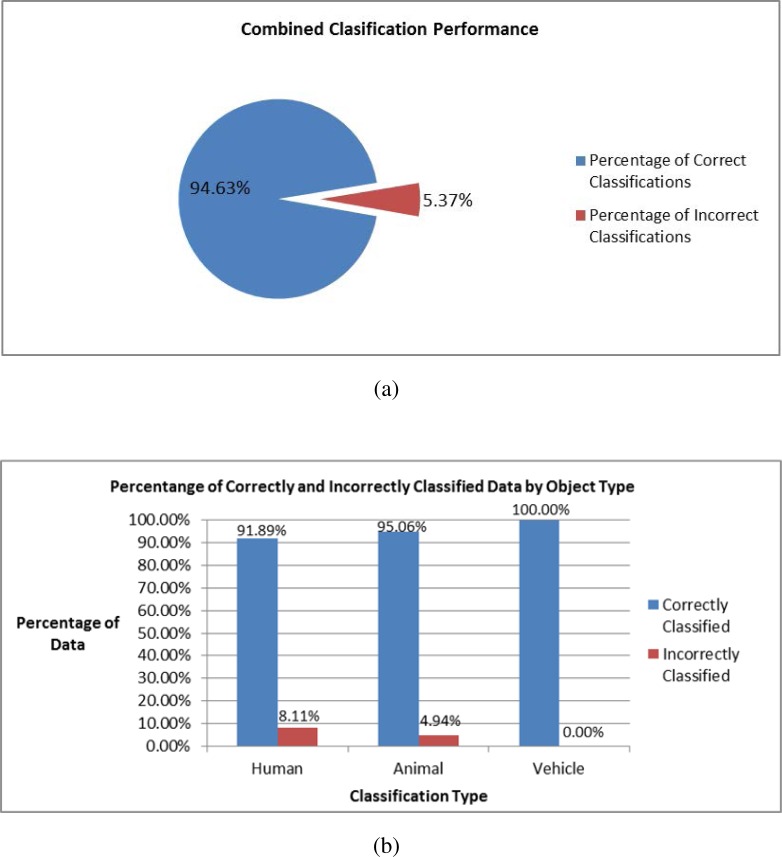
Classification results (**a**) Overall combined percent of correct and incorrect classification results, (**b**) Correct and incorrect classification results by object classes.

The neural network takes the binary values in the input layer to determine the result, shown in the output layer as positive real numbers between 0 and 1; the output processing element with the highest value indicates the classification of the input. Each test data sample was fed through the input layer of the neural network. The sequence of linear operations and activation functions used by each processing element are discussed in Section 2.2.5. These values are passed from one layer to the next until reaching the output layer, which indicates the processing element with the highest value and corresponds to a given classification (*i.e.*, human, animal, or vehicle). The distribution of correctly and incorrectly classified test samples from each class analyzed is shown in Figure 15. The neural network classified 577 samples from the pre-existing library with 94% accuracy (Figure 16). Finally, the confusion matrix that shows the performance of the classification algorithm in this test is shown in Table 2.

Recall, the classification results summarized in Figure 16 are when using the library generated by the first N-IR profiling sensor and not data acquired by the improved wireless profiling sensor. Building a comprehensive library with the wireless profiling sensor is future work and out of the scope of this paper. Nevertheless, the new wireless profiling sensor was used to collect and classify 164 humans with 95.7% accuracy (with 7 objects incorrectly classified as animal). This preliminary data acquired directly from the wireless sensor has comparable accuracy to when using the original library. It is important to point out that constructing an object’s binary silhouette by the time segmentation method in Section 2.2.3 requires receiving two change-of-state packets. Therefore, to prevent packet collision resulting in data loss, the wireless prototype detectors were arranged with small horizontal offsets of approximately 5–10 inches prior to data collection. Decreasing the horizontal offsets would likely increase the accuracy of the classification above 94% given that the neural network was trained with a pre-existing library from sensors with no horizontal offsets.

Therefore, for new configurations of the wireless N-IR profiling sensors detectors, where significant horizontal offsets are anticipated, it would be necessary to retrain the neural network with data collected from the new detector configurations, that is, from the new placements of the sensor nodes that comprise the overall wireless profiling sensor. Rapidly training the neural network given a wide-variety of sensor node placements, including *ad hoc* placements, is of very high interest and the subject of future work. A current limitation of the existing wireless sensor design, including the neural network used for object classification, is that passing objects of the same type must have an approximately constant velocity. When a passing object changes its velocity in the horizontal space between each sensing node, the silhouette may become significantly distorted and classification results can be affected, unless a new neural network is trained to accommodate the variability of the velocity for objects of the same type.

#### 2.2.7. Deployment Issues

Having a well-concealed profiling sensor will improve its effectiveness as a security device since activities of interest are less likely to occur in known surveillance areas. Concealment is a deployment challenge for profiling sensors. The wireless prototype provides a proof of concept profiling sensor and it will enable more sophisticated options for concealment in real-world application scenarios.

Improving the battery life of wireless equipment is particularly important for profiling sensors, as these sensors will be considered as disposable in many anticipated applications. The time segmentation method was used to reduce the amount of data sent from the sensor nodes to the base station, thereby reducing power usage, and increasing battery life. However, this process can be further optimized for minimal battery consumption. For instance, the sensing nodes could collect data at a specified sampling rate and then send the binary string collected from each height to the wireless base station for its further processing. In addition, the sensing nodes could communicate with each other once, immediately following deployment, such that all nodes that comprise a given profiling sensor would have recorded their relative position. Such a dynamic scheme will be particularly important for *ad hoc* distribution of sensor nodes. These positions could be used as additional information to realign the collected data and improve classification accuracy. These additional features would make up a more efficient, robust, and easier to deploy profiling sensor.

## 3. Alternative Profiling Sensor Models

In addition to the work performed on N-IR profiling sensors, alternative profiling sensor models are currently being pursued to classify silhouettes, including a pyroelectric sensor and a 360° sensor. Both of these devices have the capability to support some of the same potential applications as the N-IR profiling sensors; however, at this time, such alternative approaches appear to be significantly more expensive than the sparse detector array profiling sensors previously discussed in Section 2. These alternative approaches typically forgo the sparse detector array for a more traditional dense focal-plane array and emulate a sparse detector array by extracting a subset of pixels of interest.

### 3.1. Linear Pyroelectric Array Profiling Sensor

The linear pyroelectric array profiling sensor is a passive system that uses long wave infrared (LWIR) detectors [2,5,21]. Unlike any of the N-IR profiling sensors described in Section 2, the pyroelectric approach does not generate radiation. Consequently, the pyroelectric sensor takes advantage of the radiation, namely heat, emitted by the objects of interest to generate a silhouette of the object in the camera’s field of view. Such techniques typically use a denser focal-plane array as compared with N-IR profiling sensors and extract a subset of pixels of interest, in effect emulating a sparse detector array. With the pyroelectric approach, a four paneled blackbody was used to calibrate the infrared camera by determining the linear relationship between the preset temperatures and grayscale values. The collection of data, in the form of two-dimensional images, required techniques to achieve image segmentation, feature extraction, and classification results according to the system specifications.

**Figure 17.**
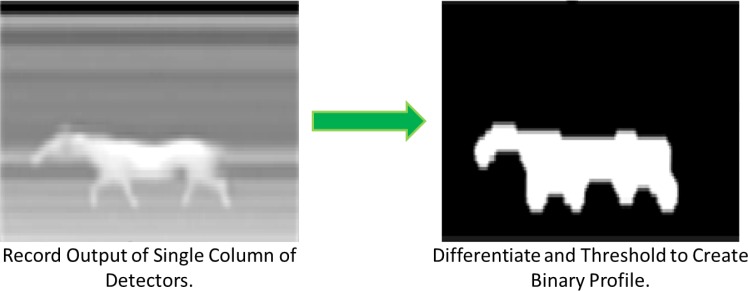
Block diagram showing the process of simulation [2].

The features of interest were first isolated and later defined through image processing. Since the background in the field of view remains stationary, it is eliminated from the image using velocity thresholding. A similar thresholding technique is used to convert the grayscale image to a binary black and white image. Figure 17 shows a block diagram of the profile generation process leading to the binary image used for classification from a passive IR sensor that emulates a profiling sensor by extracting a single column of detectors [2].

Several classification algorithms including a Naïve Bayesian, Naïve Bayesian with Linear Discriminant Analysis for dimensionality reduction, K-Nearest Neighbor, and Support Vector Machines were compared with study their performance classifying human and animals [2]. Brown *et al.*[21] use a Naïve Bayesian classification method that operates over “reasonably complete” binary images. The selected images under this category are binary images that contain sufficient features that can be used to easily identify the object in the image as a human or animal by the naked eye. Examples of these features are the head and arms of a human, which make the object in the image easily identifiable as a human. The same approach would apply for an animal for assignment into the animal classification. This method is highly successful at classifying passing objects, both in the laboratory and in the field as shown by Brown *et al.*[21], Chari *et al.*[2], and White *et al.*[5].

### 3.2. 360° Profiling Sensor

The 360° profiling sensor is a non-sparse detector that uses a long wave infrared camera focused on a conical mirror to achieve a 360° field of view [28]. The conic mirror provides continuous field of view coverage in the horizontal regions but its size and shape constrains coverage in the vertical direction, though the cones are interchangeable. Since the maximum height of a target can be estimated and the design of the conic mirror can be changed to accommodate such vertical heights, full vertical coverage is unnecessary for anticipated applications.

(11) α=90°−2ϕ

(12) $$\beta =2\phi -90{}^{\circ}+\frac{{\mathrm{FOV}}_{m}}{2}$$

(13) $$\phi =\text{arctan}\left(\frac{h_{c}}{r_{b}}\right)$$

**Figure 18.**
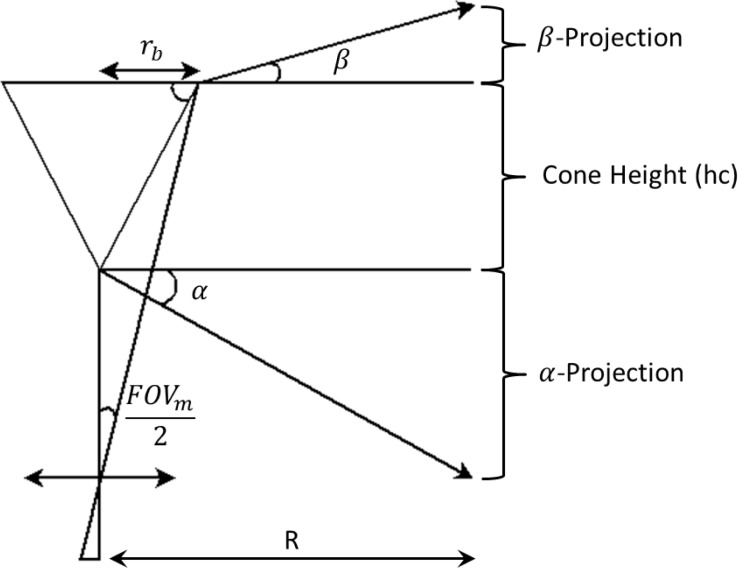
Layout of a 360° profiling sensor [28].

**Figure 19.**
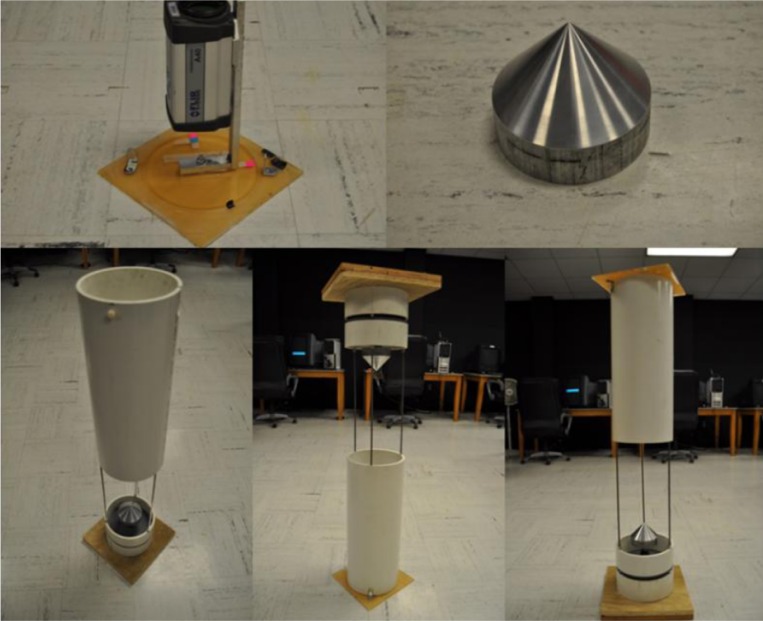
360° profiling sensor prototype designed and built at the University of Memphis [28].

Figure 18 shows how the projection angles *α* and *β* can be calculated by using Equations (11) and (12), where *FOV_m_* is the minimum field of view of the camera covered by the concentric conic mirror [28]. Equation (13) computes the internal angle of the cone base, *ϕ*, where *h_c_* and *r_b_* are the height of the cone and the radius of the base of the cone, respectively. Theoretically, these equations can be used to design a conic mirror to cover a new field of view, this being narrower or wider depending on the application of interest. However, different designs may distort the image further, thus making the segmentation, feature extraction, and classification algorithms less successful. Figure 19 shows the 360° profiling sensor model designed and built in the laboratory at the University of Memphis [28].

With this conical mirror, some image processing is necessary due to the radial distortion caused by the curvature of the mirror. Although the radial aspect of the image is distorted, the vertical image remains undistorted. For an object present in the field of view of the 360° profiling sensor, several sample frames are taken and statistical analysis is used to separate the targeted object from its background. Brown *et al.*[28] explain the different statistical methods used to separate the image into the segment of interest in addition to feature extraction and classification methods.

## 4. Conclusions

As shown by the results presented here and those in related articles, sensors that classify a crude silhouette are a reliable means of surveillance, especially for broad-scale object classification into classes such as human, animal or vehicle. Several versions of profiling sensors have been developed, including N-IR, pyroelectric, and 360° profiling sensors. Though each type of sensor operates differently to obtain the initial silhouette or profile, they all can function as a form of profiling sensor. The N-IR sensors, which utilize a sparse detector array, have evolved more rapidly than alternative approaches and appear to be a lower-cost option, which will be critical in application scenarios in which the sensors must be regarded as disposable.

This paper advanced work in N-IR profiling sensors by realizing the sparse detector array as a collection of wireless sensor nodes, whose data can be aggregated at a base station to realize a profiling sensor. This advancement is significant as it facilitates *ad hoc* deployment options for a profiling sensor’s detectors, including scenarios in which the detectors must be remotely and randomly deployed in network-centric sensing environments for intelligent fence and analogous applications.

A back-propagation neural network was also developed for the wireless version of the N-IR profiling sensor, with initial results that show classification accuracy of sensed objects into the broad categories of human, animal or vehicle at approximately 94%. It is anticipated that this classification accuracy will be increased by extensive training of the neural network via data acquired from the exact deployment configuration of the wireless sensor’s detectors or nodes, rather than using data from a pre-existing library.

This paper advances profiling sensors toward a more easily deployable, concealable, and energy efficient design. Nevertheless, additional enhancements of the wireless prototype will be necessary before it is ready for practical deployment, including improved packaging of the sensor node to withstand anticipated environmental conditions of the deployment site. Moreover, the sensor node to sensor node communication to dynamically determine the relative locations of each node will enhance the ad hoc sensor deployment and the training of the back-propagation neural network.

## Figures and Tables

**Table 1.** Number of samples per class used in the neural network training and testing processes.

	**Training**	**Testing**
**Human**	337	333
**Animal**	81	81
**Vehicle**	162	163

**Table 2.** Confusion matrix of classification results.

		**Predicted Class**		
		Human	Animal	Vehicle
**Actual Class**	Human	306	20	7
	Animal	4	77	0
	Vehicle	0	0	163
